# New Strain of Influenza A Virus (H5N1), Thailand

**DOI:** 10.3201/eid1303.061103

**Published:** 2007-03

**Authors:** Salin Chutinimitkul, Thaweesak Songserm, Alongkorn Amonsin, Sunchai Payungporn, Kamol Suwannakarn, Sudarat Damrongwatanapokin, Arunee Chaisingh, Bandit Nuansrichay, Thaweesak Chieochansin, Apiradee Theamboonlers, Yong Poovorawan

**Affiliations:** * Chulalongkorn University, Bangkok, Thailand; † Kasetsart University, Nakorn Pathom, Thailand; ‡ National Institute of Animal Health, Bangkok, Thailand

**Keywords:** H5N1, influenza A virus, vaccine, letter

**To the Editor:** During 2004–2005, 3 major waves of avian influenza outbreaks occurred in Thailand (*1*). The first wave was reported in early January 2004, the second in July 2004, and the third in October–December 2005. In total, 22 persons were infected and 14 died. Recently, a fourth wave began on July 23, 2006. The Thai Ministry of Public Health reported that avian influenza A (H5N1) virus killed 2 infected persons. The first patient, a 17-year-old man in Phichit Province, began to experience symptoms on July 15, 2006, and died on July 24, 2006 (*2*). The second patient, a 27-year-old man in Uthai Thani Province, began to experience symptoms on July 24, 2006, and died on August 3, 2006 (*3*).

The fourth wave of these outbreaks involved chickens and encompassed 2 distinct areas: Phichit Province, identified on July 23, 2006 (*4*), and Nakhon Phanom Province, identified on July 28, 2006 (*5*). We sequenced all 8 gene segments of the 2 viruses isolated from Phichit and 1 virus isolated from Nakhon Phanom and then submitted to GenBank as follows: A/chicken/Thailand/PC-168/2006 (DQ999879–86) and A/chicken/Thailand/PC-170/2006 (DQ999887–94) from Phichit and A/chicken/Thailand/NP-172/2006 (DQ999871–8) from Nakhon Phanom.

Whole genome analysis showed that all 3 samples had undergone minor mutations that are typical of circulating influenza A viruses. Unexpectedly, this outbreak was associated with 2 strains of the virus. The 2 samples from Phichit closely resembled H5N1 strains that had circulated in Thailand during 2004 and 2005. The sample from Nakhon Phanom was newly observed in Thailand and more closely related to H5N1 strains that had been circulating since 2005 in southeast People’s Republic of China. The whole genome phylogenetic analysis also showed that the viruses isolated from Phichit belonged to genotype Z, whereas virus isolated from Nakhon Phanom belonged to genotype V, which differs from genotype Z in the PA gene (*6*) (Figure, panel A).

**Figure:**
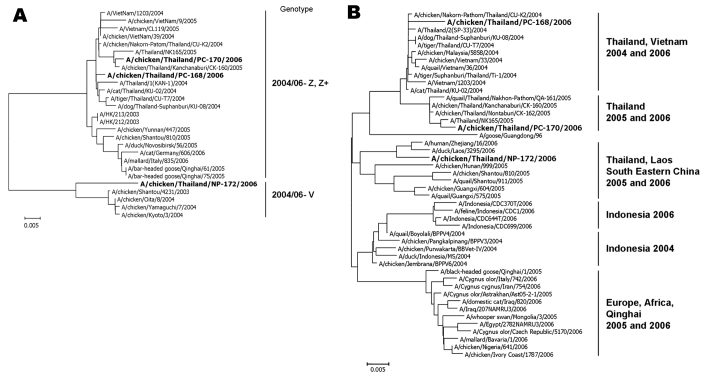
A) Phylogenetic relationships of the polymerase acid protein gene comparing genotype Z, Z+, and V. B) Hemagglutinin gene of influenza A (H5N1) viruses in Thailand 2006 compared with several H5N1 strains worldwide. For a larger reproduction of the phylogenetic relationships.

The phylogenetic tree of the hemagglutinin (HA) gene (Figure, panel B) showed that the Phichit samples were similar to the cluster of samples isolated during 2004 and 2005 in Thailand and Vietnam. In contrast, the Nakhon Phanom sample was clustered into the same group with viruses isolated from southeast People’s Republic of China, including Zhejiang, Shantou, Hunan, Fujian, Guangxi, and Lao People’s Democratic Republic (*7*) with the differences in the cleavage site, SPLRERRRK-R/G (underline and dash indicate differences), which had never been found in Thailand. The N-link glycosylation sites (positions 154–156) of the Pichit isolates were NST residues, whereas in the Nakhon Phanom isolate, NNT residues were observed. However, the receptor-binding site of HA (positions 222 and 224) was unchanged.

In the neuraminidase (NA) gene, the new isolates contain 20 amino acid deletions within the stalk region, the same as previously described (*1*). The ESEV residues in the C-terminal and Asp92 of NS1 were observed in the 2006 isolates and in viruses that have been isolated from Thailand, Vietnam, and People’s Republic of China. This finding indicates that the new isolates were highly virulent but sensitive to treatment with interferon and tumor necrosis factor-α (*8*). The 2006 isolates contain Glu627 of PB2, identical to the previous isolates from Thailand and Indonesia, which may indicate that the new isolates had less efficient replication capability in mammalian hosts (*9*). Drug resistance or sensitivity is based on sequences of M2 and NA. Substitution within residues including L26I, V27A/I, A30S, and S31N of the M2 ion channel protein was used to predict amantadine-resistant mutants, and H274Y of the NA was used to predict for oseltamivir resistance (*10*). The virus observed in 2006 isolates from Phichit was resistant to amantadine but sensitive to oseltamivir, whereas the isolate from Nakhon Phanom was sensitive to amantadine and oseltamivir, which implies that infected patients received different antiviral drugs.

According to previous World Health Organization reports, the HA sequences of most influenza (H5N1) viruses that circulated in avian species during the past 3 years are separated into 2 distinct phylogenetic clades. Clade 1 viruses that circulated in Cambodia, Thailand, and Vietnam were responsible for human infections in those countries during 2004 and 2005. Clade 2 viruses that circulated in birds in People’s Republic of China and Indonesia during 2003–2004 and 2005–2006 spread westward to the Middle East, Europe, and Africa. This latter genetic group of viruses has been principally responsible for human infections during late 2005 and 2006 (*11*). The latest wave of the outbreaks in Thailand was caused by viruses closely related to those that caused outbreaks in Thailand in 2004–2005 and to viruses recently circulating in southeast People’s Republic of China and other Southeast Asian countries. This finding raises concern for development of new candidate influenza (H5N1) vaccine strains. Geographic spreading, epidemiology, and genetic properties of recently circulating influenza (H5N1) viruses should be considered when developing candidate H5N1 strains of influenza vaccine.
